# Synthesis and Promotion of the Osteoblast Proliferation Effect of Morroniside Derivatives

**DOI:** 10.3390/molecules23061412

**Published:** 2018-06-11

**Authors:** Hua Han, ZhengQing Li, Na Qu, Si Chen, PeiLiang Dong

**Affiliations:** Key Laboratory of Chinese Materia Medica, Ministry of Education, Heilongjiang University of Chinese Medicine, Harbin 150040, China; hh7551@163.com (H.H.); lizhengqing1125@163.com (Z.L.); Qu399127818@163.com (N.Q.); m13945033486@163.com (S.C.)

**Keywords:** morroniside, derivatization, osteoblast proliferation effect, MC3T3-E1

## Abstract

*Sambucus williamsii* Hance has been used in fractures for thousands of years, but research on its active components, such as morroniside, until now had not been carried out. In this study, morroniside was taken as the leading compound, and fourteen derivatives were synthesized. The promotion of osteoblast proliferation effect of the derivatives was evaluated on MC3T3-E1 cells. Five derivatives (**2**, **3**, **4**, **5**, and **14**) showed a good proliferation effect on MC3T3-E1 cells, and their promoted expression effects on OC (Osteocalcin) and ALP (Alkaline phosphatase) in MC3T3-E1 cells were measured. Compound **3** was shown to have the strongest proliferation effect (EC_50_ = 14.78 ± 1.17 μg/mL) and to significantly promote the expression of OC and ALP.

## 1. Introduction

Fracture is one of the most common clinical diseases and often causes substantial damage to a patient’s quality of life [1]. Without proper care and recovery, it can cause irreversible damage to the patient [2]. In recent years, with the increase in the elderly population, the incidence of osteoporosis has become the seventh most common affliction in the world, and about 80% of elderly people have different degrees of osteoporosis [3,4]. Due to osteoporosis in patients with osteopenia, bone strength decreases, bone fragility increases, and the probability of fracture greatly increases [5]. Moreover, the healing process of osteoporosis fracture is long, and clinical efficacy is extremely low, seriously affecting the daily life of the elderly [6].

Currently, conservative treatment of osteoporotic fracture in the elderly has become one of the most commonly used clinical methods [7]. The main drugs for the treatment of fractures in clinics are anti-inflammatory drugs, analgesics, and drugs that promote fracture healing [8,9]. *Sambucus williamsii* Hance has been used in the clinical treatment of various types of fractures for thousands of years, with short course, rapid recovery, and high efficacy [10]. Previous studies have shown that morroniside is the main component of *S. williamsii* Hance [11,12], which can lower serum calcium, promote the deposition of calcium salts, promote collagen synthesis, improve the quality of callus, and has great potential for clinical applications [13,14,15]. However, our previous studies have shown that morroniside, when used alone, weakly promotes the proliferation of MC3T3-E1 cells (the proliferation rate is about 20% in 24 h with 62.5 μg/mL morroniside) [15,16]. In addition, the poor solubility, the rapid metabolism in the body, and the low bioavailability of morroniside has limited its clinical application, so morroniside cannot be developed as a clinical drug [17].

In this study, we used morroniside as a leading compound to obtain different derivatives to obtain a compound with more value and a strong promoting effect on fracture healing. Two of the derivatives are new compounds (**12** and **13**).

## 2. Results and Discussions

### 2.1. Preparations of Morroniside Derivatives

**Figure 1:**
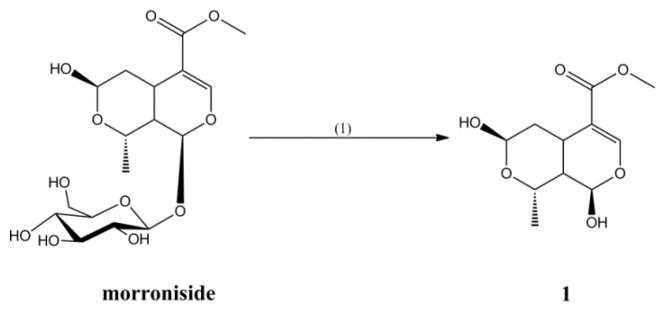
Synthesis of Compound **1**. (1) 20% HCl, 50 °C, 1 h.

Compound **1** was synthesized by hydrolyzing morroniside in a 20% hydrochloric acid aqueous solution at 50 °C for 1 h, in a yield of 35% (Figure 1).

**Figure 2:**
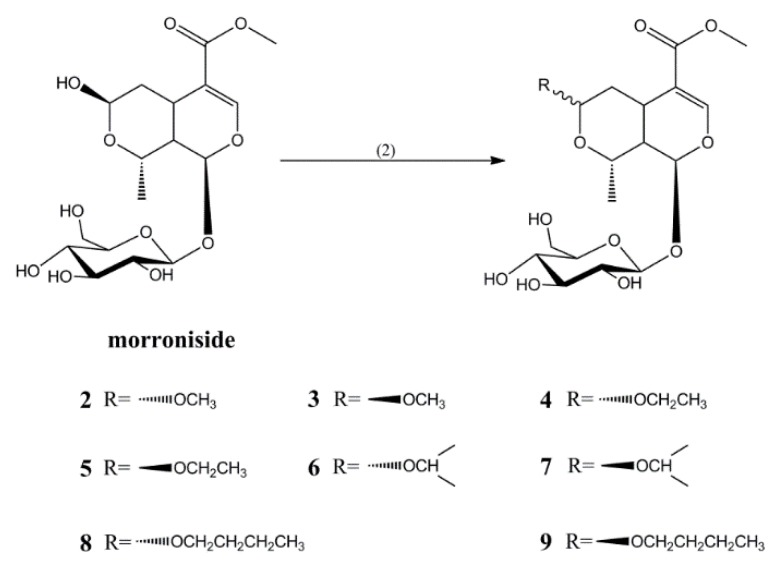
Synthesis of Compounds **2**–**9**. (2) RH, concentrated nitric acid, r.t., 30 min.

Compounds **2**–**9** were synthesized by reacting morroniside with different alcohols at room temperature in the presence of concentrated nitric acid as a catalyst for 30 min, for yields of 23%, 55%, 26%, 51%, 24%, 48%, 24%, and 49%, respectively (Figure 2).

**Figure 3:**
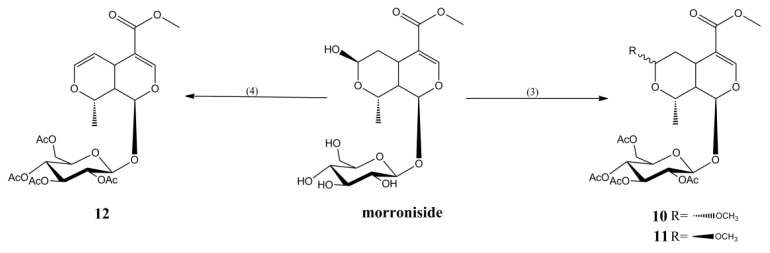
Synthesis of Compounds **10**–**12**. (3) MeOH, acetic anhydride, 80 °C, 1 h. (4) MeOH, acetic anhydride, 200 °C, 1 h.

Compounds **10**–**12** were synthesized by reacting morroniside with acetic anhydride at different temperatures in methanol for 1 h, for yields of 22%, 40%, and 45%, respectively (Figure 3).

**Figure 4:**
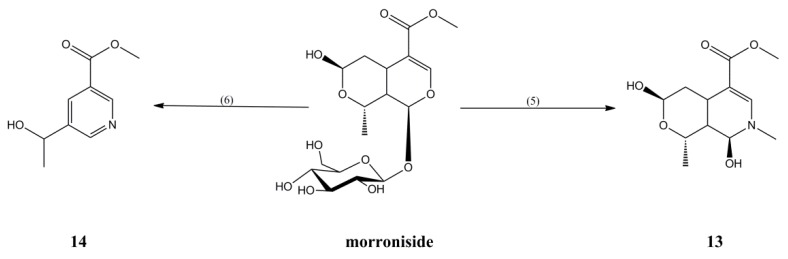
Synthesis of Compounds **13** and **14**. (5) Methylamine ethanol solution, 10 °C, 3 h. (6) C_2_H_5_OH, HOAc-NaAc buffer solution, β-glucosidase, 50 °C, 3 h.

Compounds **13** and **14** were synthesized as monoterpene alkaloids derivatives with different primary amines, in yields of 32% and 46%, respectively (Figure 4).

### 2.2. Cell Viability Assay

The MTT assay was used to evaluate the proliferation effect of the morroniside derivatives on MC3T3-E1 cells, and their values EC_50_ are shown in Table 1. Five compounds (**2**, **3**, **4**, **5**, and **14**) promoted proliferation on MC3T3-E1 cells (EC_50_ < 500) more strongly than did the other derivatives. Among these five compounds, Compound **3** showed the strongest proliferation effect on MC3T3-E1 cells, Compound **14** showed the weakest, and Compounds **2**, **5**, and **4** showed a medium effect.

The activity of morroniside is conspicuously decreased when morroniside is hydrolyzed into aglycone (Compound **1**). In addition, iridoids cannot keep in a form of prototype drug in the human body due to rapid metabolism [18], possibly resulting in low activity of morroniside in vivo.

In the case of acetylation into Compounds **10**, **11**, and **12**, morroniside gains less bioactivity. Nonetheless, the acetyl group can be removed through metabolism of enzymes, thereby releasing the morroniside and postponing its metabolism, consequently prolonging drug action time [19]. Therefore, the bioactivity of acetylated morroniside still needs to be further researched.

The activity of morroniside, to some extent, is likely to be enhanced when morroniside is alkylated at C-7 position, and enhancement only occurs when substituted with methyl or ethyl. Research also shows that the activity of morroniside will be gradually decreased with the increasing number of carbon atoms on the alkyl chain. When the number of carbon atoms on the alkyl chain exceeds 3, the activity of alkylated morroniside will be far lower than that of morroniside itself. Moreover, the activity will be tremendously enhanced if the substituent is in β configuration rather than α configuration.

Research on alkaloids has been extensively carried out owing to the diverse structural characteristics and pharmacological actions of such substance [20]. In this study, when aglycone is transformed into pyridine monoterpene alkaloids derivatives (Compound **13**), the pharmacological activity is significantly enhanced, which proves that alkaloids have massive pharmacological potential. Besides, the activity of morroniside is conspicuously stimulated when its mother nuclear structure is broken, which transforms morroniside into Compound **14**. Furthermore, the stability of alkaloids is stronger than that of morroniside because glycosidic linkage and hemiacetal structure are absent in Compounds **13** and **14**. It is speculated that the metabolism of alkaloids probably takes much longer time than that of morroniside and its action efficacy is likely to be further extended as a consequence.

### 2.3. Activities of OC and ALP Assay

**Figure 5:**
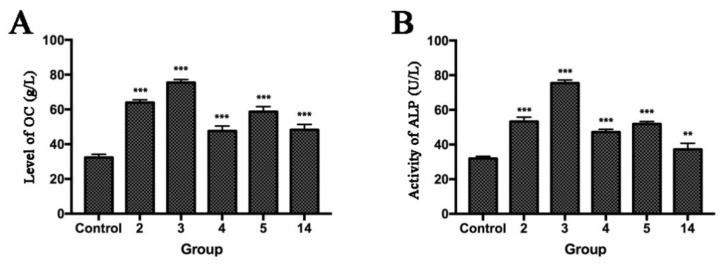
Promotion effect of 90 μg/mL of **2**, **3**, **4**, **5**, and **14** on OC and ALP expression in MC3T3-E1 cells: (**A**) OC levels; and (**B**) ALP activities. All values are represented as mean ± SD, *n* = 5. ** *p* < 0.01 versus the control. *** *p* < 0.001 versus the control.

Osteocalcin (OC) and alkaline phosphatase (ALP) are two substances secreted by osteoblasts [21,22] and can promote the deposition of bone salt. The expression of OC and ALP can reflect the rate of fracture healing [23]. Therefore, in this study, we measured the effects of five compounds on the expressions of OC and ALP in osteoblasts, and the results are shown in Figure 5. 

As can be seen in the figure, the above-mentioned five derivatives are able to elevate the expression OC and ALP, in significant contrast to the blank group (*p* < 0.05). Among the five derivatives, Compound **3** stands out as possessing remarkable efficacy superior to the other four (*p* < 0.05). It is shown that Compound **3** has the best activity in elevating the expression OC and ALP.

## 3. Materials and Methods

### 3.1. General

NMR spectra were performed on a Bruker DRX-400 NMR spectrometer at 400 MHz using CD_3_OD as solvent in Zhengzhou University. The purity of morroniside and the derivatives were checked on a Waters e2695 liquid chromatograph equipped with 2424 ELS detector and 2998 PDA detector. GF254 silica plate was purchased from Qingdao Haiyang Chemical Co. Ltd. (Qingdao, China). The Elisa kits of OC (H152) and ALP (A059-2) were purchased from Nanjing Jiancheng Bioengineering Institute (Nanjing, China). All the chemical reagents were purchased from Sinopharm Chemical Reagent Co., Ltd. (Shanghai, China). The morroniside was extracted and purified in the medicinal chemistry department, Heilongjiang University of Chinese Medicine.

### 3.2. Synthesis

#### 3.2.1. Synthesis of Compound **1**

An amount of 0.1 g of morroniside was dissolved with 2 mL of a 20% hydrochloric acid solution, and the mixture was heated for 1 h at 50 °C. After this mixture was cooled to room temperature, saturated sodium bicarbonate was added to remove the hydrochloric acid, the solvent was recovered in vacuum, and the residue was purified by silica gel column chromatography and eluted with petroleum ether-ethyl acetate (5:1) to obtain Compound **1** (21.1 mg, 35% yield).

The ^1^H-NMR and ^13^C-NMR results of Compound **1** are consistent with β-morroniside aglycone, so it was concluded that Compound **1** is β-morroniside aglycone [24].

#### 3.2.2. Synthesis of Compounds **2**–**9**

An amount of 0.1 g of morroniside was dissolved with 2 mL of different alcohols (methanol, ethanol, isopropanol, and *n*-butanol), and 0.1 mL of concentrated nitric acid was added. This mixture was stirred for 30 min at room temperature. After the reaction, moderate saturated sodium bicarbonate was added to remove the concentrated nitric acid, the solvent was recovered in vacuum, and the residue was purified by silica gel column chromatography and eluted with dichloromethane-methanol (16:1) to obtain Compounds **2** (23.8 mg, 23% yield), **3** (56.9 mg, 55% yield), **4** (27.8 mg, 26% yield), **5** (54.5 mg, 51% yield), **6** (26.5 mg, 24% yield), **7** (53.0 mg, 48% yield), **8** (27.3 mg, 24% yield), and **9** (55.8 mg, 49% yield).

The ^1^H-NMR and ^13^C-NMR of Compounds **2**–**9** are consistent with those reported in the previous study. However, it is worth noting that, if the configuration at C-7 is α, the chemical shift of C-5 is more than 30, and C-8 is more than 70; if the configuration at C-7 is β, the chemical shift of C-5 is between 25 and 29, and C-8 is between 62 and 66 [25].

#### 3.2.3. Synthesis of Compounds **10** and **11**

An amount of 0.1 g of morroniside was dissolved with 2 mL of methyl alcohol, and 2 mL of acetic anhydride was added. The mixture was refluxed for 1 h at 80 °C. The solvent was recovered in vacuum, and the residue was purified by silica gel column chromatography and eluted with petroleum ether-ethyl acetate (3:1) to obtain Compounds **10** (31.9 mg, 22% yield) and **11** (58.0 mg, 40% yield).

The ^1^H-NMR and ^13^C-NMR of Compounds **10** and **11** are consistent with those reported in the previous study, so it was concluded that Compounds **10** and **11** are 7α-methoxy tetraacethyl morroniside and 7β-methoxy tetraacethyl morroniside, respectively [26].

#### 3.2.4. Synthesis of Compound **12**

An amount of 0.1 g of morroniside was dissolved with 2 mL of methyl alcohol, and 2 mL of acetic anhydride was added. The mixture was refluxed for 1 h at 200 °C. The solvent was recovered in vacuum, and the residue was purified by silica gel column chromatography and eluted with petroleum ether-ethyl acetate (3:1) to obtain Compound **12** (61.6 mg, 45% yield).

*6,7-double bond tetraacethyl morroniside* (**12**): white powder; ^1^H-NMR (400 MHz, CD_3_OD) δ: 1.44 (3H, d, *J* = 6.9 Hz, H-10), 1.97 (3H, s, 6′-CH_3_), 2.0 (1H, m, H-9), 2.01, 2.01, 2.04 (each 3H, s, 3′, 4′ and 2′-CH_3_), 3.41 (1H, m, H-5), 3.71 (3H, s, H-12), 3.92 (1H, m, H-2′), 4.14 (1H, dd, *J* = 2.5 Hz, 12.3 Hz, H-6′a), 4.18 (1H, o, H-5′), 4.31 (1H, dd, *J* = 4.9 Hz, 17.3 Hz, H-6′b), 4.69 (1H, dt, *J* = 1.6 Hz, 6.2 Hz, H-6), 4.96 (1H, dd, *J* = 8.1 Hz, 9.7 Hz, H-4′), 5.03 (1H, t, *J* = 6.9 Hz, H-8), 5.15 (1H, d, *J* = 8.1 Hz, H-1′), 5.33 (1H, dd, *J* = 9.5 Hz, 19.0 Hz, H-3′), 5.40 (1H, d, *J* = 8.6 Hz, H-1), 6.30 (1H, dd, *J* = 2.3 Hz, 6.1 Hz, H-7), 7.48 (1H, s, H-3); ^13^C NMR (100 MHz, CD_3_OD) δ:19.5, 20.5, 20.6, 20.6, 20.6, 31.0, 39.0, 51.9, 62.9, 69.9, 72.5, 73.1, 74.1, 74.8, 96.5, 98.7, 103.8, 110.3, 144.1, 153.2, 168.7, 171.1, 171.3, 171.6, 172.3; ESI-MS *m*/*z* 557 [M + H]^+^ (calcd for C_25_H_32_O_14_).

#### 3.2.5. Synthesis of Compound **13**

An amount of 0.1 g of morroniside was dissolved with 2 mL of a methylamine alcohol solution, and the mixture was stirred for 3 h at 10 °C. The solvent was recovered in vacuum, and the residue was purified by silica gel column chromatography and eluted with petroleum ether-ethyl acetate (1:1) to obtain Compound **13** (20.3 mg, 32% yield).

*2-N-Methyl morroniside aglycone* (**13**): white powder; ^1^H-NMR (400 MHz, CD_3_OD) δ: 1.30 (3H, d, *J* = 6.5 Hz, H-10), 1.50 (1H, m, H-9), 2.40 (2H, m, H-6), 2.93 (1H, m, H-5), 3.12 (3H, s, N-CH_3_), 3.67 (3H, s, H-12), 4.16 (1H, dd, *J* = 6.5 Hz, 13.9 Hz, H-8),4.72 (1H, d, *J* = 2.2 Hz, H-7), 5.10 (1H, s, H-1), 7.29 (1H, s, H-3); ^13^C-NMR (100 MHz, CD_3_OD) δ: 19.4, 24.9, 34.2, 39.2, 40.8, 51.4, 71.0, 80.4, 87.5, 104.7, 144.1, 169.9; ESI-MS *m*/*z* 258 [M + H]^+^ (calcd for C_12_H_19_NO_5_).

#### 3.2.6. Synthesis of Compound **14**

An amount of 0.1 g of morroniside was dissolved with 0.5 mL of an ethanol solution, and 5 mL of HOAc–NaOAc buffer solution (pH 5) and 0.1 g of β-glucosidase were added. The mixture was reacted for 3 h at 50 °C. The solvent was recovered in vacuum, and the residue was purified by silica gel column chromatography and eluted with petroleum ether-ethyl acetate (5:1) to obtain Compound **14** (20.5 mg, 46% yield).

The ^1^H-NMR and ^13^C-NMR of Compound **14** are consistent with those reported in the previous study, so it was concluded that Compound **14** is 5-(1′-hydroxyethyl) nicotinic acid methyl ester [27].

### 3.3. Biological Assays

The MC3T3-E1 cell line was obtained from Procell (Wuhan, China) and was grown in DMEM medium supplemented with 10% fetal bovine serum and a 1% penicillin–streptomycin solution.

The cell viability was evaluated using an MTT assay. The cells were plated in a 96-well plate with 100 μL in each well (1 × 10^4^ cells/well) and then incubated for 24 h at 37 °C. The cells were then exposed to different concentrations (100, 10, 1, and 0.1 μg/mL) of the derivatives and incubated for another 24 h, and 20 μL MTT regents (5 mg/mL) were subsequently added to each well, which was followed by incubation for 4 h. The medium was removed, and 100 μL of DMSO was added to each well, which were then shaken for 10 min. The absorbance was measured by a microplate reader at a wavelength of 490 nm. Control wells received only the medium without the derivatives. Each derivative concentration had five replicates. The cell viability was calculated using the following formula:

cell viability (%) = (A_Sample_/A_Control_ − 1) × 100%.

All results are expressed as mean ± SD. All figures were illustrated using Prism 7 (SOFTHEAD, Shenzhen, China). Statistical analysis was carried out with SPSS 24 software (STRONG-VINDA, Beijing, China).

### 3.4. Activities of OC and ALP Assays

The Elisa kits of OC (H152) and ALP (A059-2) were purchased from Nanjing Jiancheng Bioengineering Institute (Nanjing, China).

The cells were plated on a 12-well plate with 1000 μL in each well (1 × 10^4^ cells/well) and then incubated for 24 h at 37 °C. The cells were then exposed to compound **2**, **3**, **4**, **5**, and **14** in 90 μg/mL amounts, and incubated for three days. The cells and the medium were transferred to a 1.5 mL centrifuge tube, frozen and thawed three times, then placed in an ice box, and broken by an ultrasonic protein breaker. According to the ELISA kit instructions, 5 μL of double distilled water (blank), 5 μL of a 0.1 mg/mL phenol standard application solution (standard), and 5 μL of derivative solution (measurement) were added. Afterwards, 50 μL of buffer and matrix solution each were added, and the mixture was then placed in a 37 °C water bath for 15 min. An amount of 150 μL of a chromogenic agent was added, and the OD (optical density) values were measured at 520 nm.

## 4. Conclusions

Serial morroniside derivatives were synthesized, and their proliferation effects on MC3T3-E1 cells were evaluated in vitro. From the above studies, we can draw the following conclusions: (1) A series of products with alkylation at the C-7 position was synthesized by a new method. Compared with previous iodine catalysis [22], this synthetic method is more secure and economical and simplified the post-processing program. (2) Among the morroniside derivatives, Compounds **2**, **3**, **4**, **5**, and **14** had strong proliferation effects on MC3T3-E1 cells. Among them, Compound **3** exhibited the strongest effect. (3) The introduction of methyl, such as in Compounds **2** and **3**, at Position 7 improved the proliferation effect on MC3T3-E1 cells. However, with the extension of the carbon chain, the activity decreases gradually. (4) With substitution with beta configuration at Position 7, the effect of the proliferation is higher than the alpha configuration substitution, such as Compounds **2**, **3**, **4**, and **5**. (5) Acetylation in morroniside did not affect the proliferation effect on MC3T3-E1. (6) Transformation into the *N*-containing derivative (Compound **14**) slightly increases the effect, but this effect is still less substantial than the alkylation derivatives (Compounds **2** and **3**).

## Figures and Tables

**Table 1:** EC_50_ values of derivatives promoting proliferation on MC3T3-E1 cells (mean ± SD), *n* = 5.

Compound	EC_50_ (μg/mL)	Compound	EC_50_ (μg/mL)
**1**	2042 ± 3.310	**8**	1068 ± 3.029
**2**	118.2 ± 2.073	**9**	656.0 ± 2.817
**3**	14.78 ± 1.170	**10**	961.6 ± 2.983
**4**	434.2 ± 2.638	**11**	1022 ± 3.009
**5**	164.4 ± 2.216	**12**	927.0 ± 2.967
**6**	1342 ± 3.128	**13**	637.2 ± 2.804
**7**	1028 ± 3.012	**14**	307.8 ± 2.488
